# Virulence, antibiotic resistance phenotypes and molecular characterisation of *Vibrio furnissii* isolates from patients with diarrhoea

**DOI:** 10.1186/s12879-024-09273-5

**Published:** 2024-04-19

**Authors:** Yanyan Zhou, Li Yu, Ming Liu, Weili Liang, Zhenpeng Li, Zheng Nan, Biao Kan

**Affiliations:** 1 Department of Clinical Laboratory Medicine, Beijing Friendship Hospital, Capital Medical University, Beijing, 100052 China; 2 Beijing Municipal Center for Disease Prevention and Control, Beijing, 100013 China; 3 State Key Laboratory for Infectious Disease Prevention and Control, Department of Diarrheal Diseases, Chinese Center for Disease Control and Prevention, National Institute for Communicable Disease Control and Prevention, Beijing, 102206 China

**Keywords:** *Vibrio furnissii*, Virulence genes, Antibiotic resistance, Phylogeny, Genomics

## Abstract

**Background:**

*Vibrio furnissii* is an emerging human pathogen closely related to *V. fluvialis* that causes acute gastroenteritis. *V. furnissii* infection has been reported to be rarer than *V. fluvialis*, but a multi-drug resistance plasmid has recently been discovered in *V. furnissii*.

**Methods:**

During daily monitoring at a general hospital in Beijing, China, seven *V. furnissii* strains were collected from patients aged over 14 years who presented with acute diarrhoea between April and October 2018. Genome analysis and comparison were performed for virulence and antimicrobial resistance genes, plasmids and transposon islands, together with phylogenetic analysis. Antimicrobial resistance to 19 antibiotics was investigated using the microbroth dilution method. Virulence phenotypes were investigated based on type VI secretion system (T6SS) expression and using a bacterial killing assay and a haemolysin assay.

**Results:**

Phylogenetic analysis based on single-nucleotide polymorphisms revealed a closer relationship between *V. furnissii* and *V. fluvialis* than between other *Vibrio* spp. The seven *V. furnissii* isolates were in different monophyletic clades in the phylogenetic tree, suggesting that the seven cases of gastroenteritis were independent. High resistance to cefazolin, tetracycline and streptomycin was found in the *V. furnissii* isolates at respective rates of 100.0%, 57.1% and 42.9%, and intermediate resistance to ampicillin/sulbactam and imipenem was observed at respective rates of 85.7% and 85.7%. Of the tested strains, VFBJ02 was resistant to both imipenem and meropenem, while VFBJ01, VFBJ02, VFBJ05 and VFBJ07 were multi-drug resistant. Transposon islands containing antibiotic resistance genes were found on the multi-drug resistance plasmid in VFBJ05. Such transposon islands also occurred in VFBJ07 but were located on the chromosome. The virulence-related genes *T6SS*, *vfh*, *hupO*, *vfp* and *ilpA* were widespread in *V. furnissii*. The results of the virulence phenotype assays demonstrated that our isolated *V. furnissii* strains encoded an activated T6SS and grew in large colonies with strong beta-haemolysis on blood agar.

**Conclusion:**

This study showed that diarrhoea associated with *V. furnissii* occurred sporadically and was more common than expected in the summer in Beijing, China. The antibiotic resistance of *V. furnissii* has unique characteristics compared with that of *V. fluvialis.* Fluoroquinolones and third-generation cephalosporins, such as ceftazidime and doxycycline, were effective at treating *V. furnissii* infection. Continua laboratory-based surveillance is needed for the prevention and control of *V. furnissii* infection, especially the dissemination of the antibiotic resistance genes in this pathogen.

**Supplementary Information:**

The online version contains supplementary material available at 10.1186/s12879-024-09273-5.

## Background

Members of the genus *Vibrio* are abundant in marine environments [1] and in inland rivers where seawater intrusion occurs [2]. The levels of *Vibrio* spp. in various seafood commodities have been reported to be significantly higher in the summer than in other seasons [3]. Under this genus, *V. furnissii* is a motile, oxidase-positive, gram-negative, halophilic bacterium with phenotypic characteristics highly similar to those of *V. fluvialis* [4]. Although *V. furnissii* is closely related to *V. fluvialis*, it differs in its ability to produce gas through carbohydrate fermentation [4]. *V. furnissii* is a potential pathogen of European eel (*Anguilla anguilla*) [5] and also one of the non-cholera *Vibrio* species pathogenic in humans that can spread through the consumption of contaminated seafood products or exposure to coastal waters [6, 7]. *V. furnissii* has been associated with outbreaks or sporadic cases of gastroenteritis with cholera-like symptoms including diarrhoea, abdominal cramps, nausea and vomiting [7, 8]. Cases of *V. furnissii* bacteraemia associated with skin lesions or cellulitis have also been reported [9, 10]. *V. furnissii* infection has been reported to be rarer than *V. fluvialis* infection. The US Centers for Disease Control and Prevention (CDC) reported only 10 isolates of *V. furnissii* from 1997 to 2008: three from blood, two from a wound and five from stool [9]. However, since 2006, 24 sequences of *V. furnissii* have been submitted to GenBank in succession, which means that *V. furnissii* infection is increasingly reported.

*V. fluvialis* infection has been reported worldwide [11–13] and shows features of multi-drug resistance [12, 14] including resistance to fluoroquinolones and β-lactam antimicrobials, *bla*_*NDM−1*_-mediated carbapenem resistance and azithromycin resistance [15–17]. *V. fluvialis* has the ability to cause epidemics, so the rapid increase in and spread of antibiotic resistance in this pathogen during the past 20 years has become a major cause of concern [18]. *V. furnissii* is phylogenetically close to *V. fluvialis*, and recently, a *mph(A)*- and *bla*_*OXA−1*_-bearing conjugative plasmid, which mediates resistance to cephalosporins and azithromycin, was also discovered in a *V. furnissii* strain isolated from hospital sewage in Zhuhai, a coastal city in China [19].

Genome analysis of *V. furnissii* NCTC11218 isolated from estuaries [20] has shown that this species has a dynamic and fluid genome that can quickly adapt to environmental perturbation and has a series of virulence-related genes, such as quorum sensing-related genes *cqsA*, *cqsS*, *luxS*, *luxU/O* and *luxP/Q*; biofilm formation-related genes *hapR* and *vpsT*; major pilin subunit-encoding gene *tcpA*; and haemolysis-related genes *rtx* and *hlyA*. These genes are also widely distributed in other pathogenic *Vibrio* spp.

In this study, we isolated seven *V. furnissii* strains from the stool samples of patients with diarrhoea; surveyed the clinical characteristics of the sampled patients; and analysed the antibiotic resistance and virulence phenotypes of, and related genes in, the isolated strains by using whole-genome sequencing.

## Materials and methods

### *V. furnissii*isolates

We collected 1,985 stool samples from patients aged over 14 years who presented with acute diarrhoea at a general hospital in Beijing, China, between April and October 2018. All of the patients completed an epidemiological questionnaire on clinical history and physical fitness. We first enriched the stool samples in an alkaline peptone water solution (Beijing Land Bridge, China) [21], then cultured 20 µL of the resulting mixture on Columbia agar (Oxoid, UK) containing 5% sheep erythrocytes and 20 µg/mL ampicillin [22] (Sigma, USA), which was a selective agar for *Aeromonas* spp., and on gentamicin selective medium (Beijing Land Bridge, China), which was widely used as a selective *Vibrios* agar in China. Next, we performed oxidase tests (BioMerieuX, France) to screen for gram-negative rod colonies on the agar plate. The oxidase test results of *Vibrio* spp., *Aeromonas* spp. and *Plesiomonas shigelloides* colonies were positive, while those of Enterobacteriaceae colonies were negative. Finally, microorganisms were identified taxonomically using a VITEK matrix-assisted laser desorption/ionisation time-of-flight mass spectrometry (MALDI-TOF MS) system (BioMerieuX, France) and the microbial identification system VITEK II (BioMerieuX, France). Per protocol, the stool samples were also simultaneously tested for *Salmonella* spp., *Shigella* spp., *Aeromonas* spp., *Plesiomonas shigelloides* and other *Vibrio* spp. All of the strains were stored in a Luria broth (LB)–glycerol mixture (80:20) at -80 °C until identification.

### Genome sequencing

The genomic DNA of the isolates was extracted using TIANamp Bacteria DNA Kit (Beijing Tiangen, China) and then sequenced using Illumina NovaSeq PE150 at Beijing Novogene Bioinformatics Technology Co., Ltd. Sequencing libraries of ∼ 350 bp were prepared using NEBNext® Ultra™ DNA Library Prep Kits and analysed for size distribution using an Agilent 2100 Bioanalyzer and quantified using real-time polymerase chain reaction. SOAP denovo [23] was used to assemble paired reads. All of the genomic sequences are available at the National Center for Biotechnology Information (NCBI; accession nos. SAMN21988700, SAMN22062944-49). Further, as VFBJ05 and VFBJ07 were found to own transposon islands, the extracted DNA of them were further subjected to 250-bp paired-end whole-genome sequencing with 150× coverage using the Nanopore sequencer. The filtered subreads were assembled using Canu v1.5 [24], and then Circlator v1.5.5 (https://github.com/sanger-pathogens/circlator) was used to cyclise the assembled genomes. Coding gene prediction was performed using Prodigal v2.6.3 [25]. Genome annotation was performed using the RAST server (https://rast.nmpdr.org/rast.cgi). The whole-genome sequences of VFBJ05 and VFBJ07 are available at the NCBI (accession nos. SAMN35555507 and SAMN35555508).

### Average nucleotide identity (ANI) analysis

ANI analysis was used to evaluate the evolutionary distance of bacteria at the genomic level based on a Perl script previously described [26]. ANI between 19 genome assemblies was calculated using pyani (https://pypi.org/project/pyani/), and strains with ANI values > 95% were considered as the same species [27, 28]. These strains included seven newly sequenced clinical isolates of *V. furnissii* and 11 reference strains of *Vibrio* from GenBank.

### Genome analysis

Prokka [29] was used to annotate genomes. The Virulence Factor Database (VFDB) [30] was used to predict virulence-related genes by BLAST+, as described in a previous study [31]. Potential antimicrobial resistance genes were predicted using the Antibiotic Resistance Genes Database [32]. Plasmids were searched through the NCBI database (https://ftp.ncbi.nlm.nih.gov/refseq/release/plasmid/) and confirmed using Platon. Insertion sequences were found using BLAST in ISfinder (https://www-is.biotoul.fr/index.php) to obtain the transposon islands.

### Genome comparison

To obtain the phylogenetic tree of *V. furnissii* strains, including seven sequences from our study and 24 available sequences from GenBank, MUMmer (Version 3.23) was first used to filter out gaps and single-nucleotide polymorphisms that were less than 5 bp long and FastTree was used to construct the tree. The phylogenetic tree and gene presence/absence profiles of 31 *V. furnissii* strains were integrated and rendered using iTOL 3 [33].

### Antibiotic susceptibility tests

Antibiotic susceptibility tests were performed using antimicrobial susceptibility testing panel for *Vibrios* (Shanghai Biofosun, China). After *V. furnissii* strains were refreshed on blood agar plates at 35 °C for 16–18 h, 0.5 McFarland standard of direct colony suspension were prepared and diluted into panel according to the manufacturer’s instructions, the panel were then incubated at 35 °C for 18–20 h. The minimum inhibitory concentrations of the following 19 antibiotics were determined according to the guidelines of Clinical and Laboratory Standards Institute [34, 35]: ampicillin, ampicillin/sulbactam, cefazolin, ceftazidime, cefepime, aztreonam, amikacin, gentamicin, ciprofloxacin, levofloxacin, imipenem, meropenem, tetracycline, doxycycline, chloramphenicol, sulphonamides, trimethoprim–sulfamethoxazole, azithromycin and streptomycin. *Escherichia coli* ATCC 25,922 was used as the quality-control strain for susceptibility testing.

### Bacterial killing assay

The assay was performed as described previously [36]. Overnight cultures of *V. furnissii* (predator) were mixed with *E. coli* MG1655 (prey) at a 5:1 (predator: prey) ratio. The mixture (5 µL) was spotted onto LB agar with a 0.22-µm filter membrane. After incubating at 30 °C for 4 h, the surviving cells of the prey MG1655 were determined by 10-fold serial dilutions on rifampin-containing LB agar plates.

### Analyses of type VI secretion system (T6SS) expression

Protein samples were prepared as previously described [37]. Overnight cultures of *V. furnissii* were inoculated 1% in fresh LB medium and grown at 30 °C until an OD_600_ of ∼ 1.0 was reached. The cultures (1 mL) were then centrifuged for 2 min at 8000 rpm, and the obtained pellets were resuspended in sodium dodecyl sulphate-loading dye and boiled for 10 min. Proteins were loaded on an SDS-PAGE gel and separated by electrophoresis, after which proteins were transferred to a polyvinylidene difluoride (PVDF) membrane (Bio-Rad). The protein-bound membrane was blocked with 5% (wt/vol) non-fat milk in PBS with Tween 20 (PBST) buffer for 2 h at room temperature. The resulting membrane was cut according to the location of interest proteins, then incubated with primary antibodies (anti-Hcp and anti-RpoB) and secondary antibodies (goat anti-rabbit IRDye and goat anti-mouse IRDye), and finally imaged by two-color infrared laser imaging system (LI-COR odyssey CLx). These antibodies were prepared as previously described [38].

### Haemolysin assay

For haemolysin assays, *V. furnissii* strains were grown to the mid-log phase at 37 °C. Portions (5 µL) of concentrated cultures were then spotted onto Columbia blood agar plates and incubated for 48 h at 30 °C [39].

## Results

### Clinical features

There were ultimately 349 diarrhoea cases related to bacterial infection, which included 145 cases caused by *Vibrio* spp. Apart from *V. parahaemolyticus*, *V. cholerae* and *V. fluvialis*, seven *V. furnissii* strains were recovered from the stool samples of the patients. Among these *V. furnissii*, three of *them* (42.9%, 3/7) was only isolated from AMP blood agar, and four of them (57.1%, 4/7) could be isolated from both AMP blood agar and gentamicin selective medium.

The clinical and epidemiological characteristics of the seven patients with diarrhoea related to *V. furnissii* infection are shown in Table 1. The sex ratio (male: female) was 2.5. Among these patients, one (14.3%) also had vomiting, two (28.6%) had abdominal pain and three (42.9%) had watery stool, but none had fever. Leukocytes were found under high magnification (×40) in the stool samples of two (28.6%) of the seven patients. These seven patients reported having had no exposure to seawater or seafood.

**Table 1:** Clinical characteristics of the seven patients with diarrhoea caused by *Vibrio furnissii* infection

Strain	Sex^a^	Age	Month	Fever^b^	Diarrhoea No./day	Abdominal pain	Stool consistency	Vomiting	Erythrocytes No./Hp^c^	Leukocytes No./Hp^c^
VFBJ01	M	45	May	-	3	-	watery	-	0	0
VFBJ02	F	65	June	-	10	-	loose	-	0	0
VFBJ03	M	15	July	-	10	-	watery	+	0	1
VFBJ04	M	62	July	-	4	-	loose	-	0	0
VFBJ05	F	36	July	-	3	+	loose	-	3	30
VFBJ06	M	64	July	-	6	-	watery	-	0	0
VFBJ07	M	72	August	-	7	+	loose	-	0	0

^a^M, male; F, female

^b^Defined as an axillary temperature > 37.7 °C

^c^The erythrocyte or leukocyte number identified in stool samples under high magnification (×40)

### General bioinformatic features of the seven *V. furnissii*strains

**Fig. 1:**
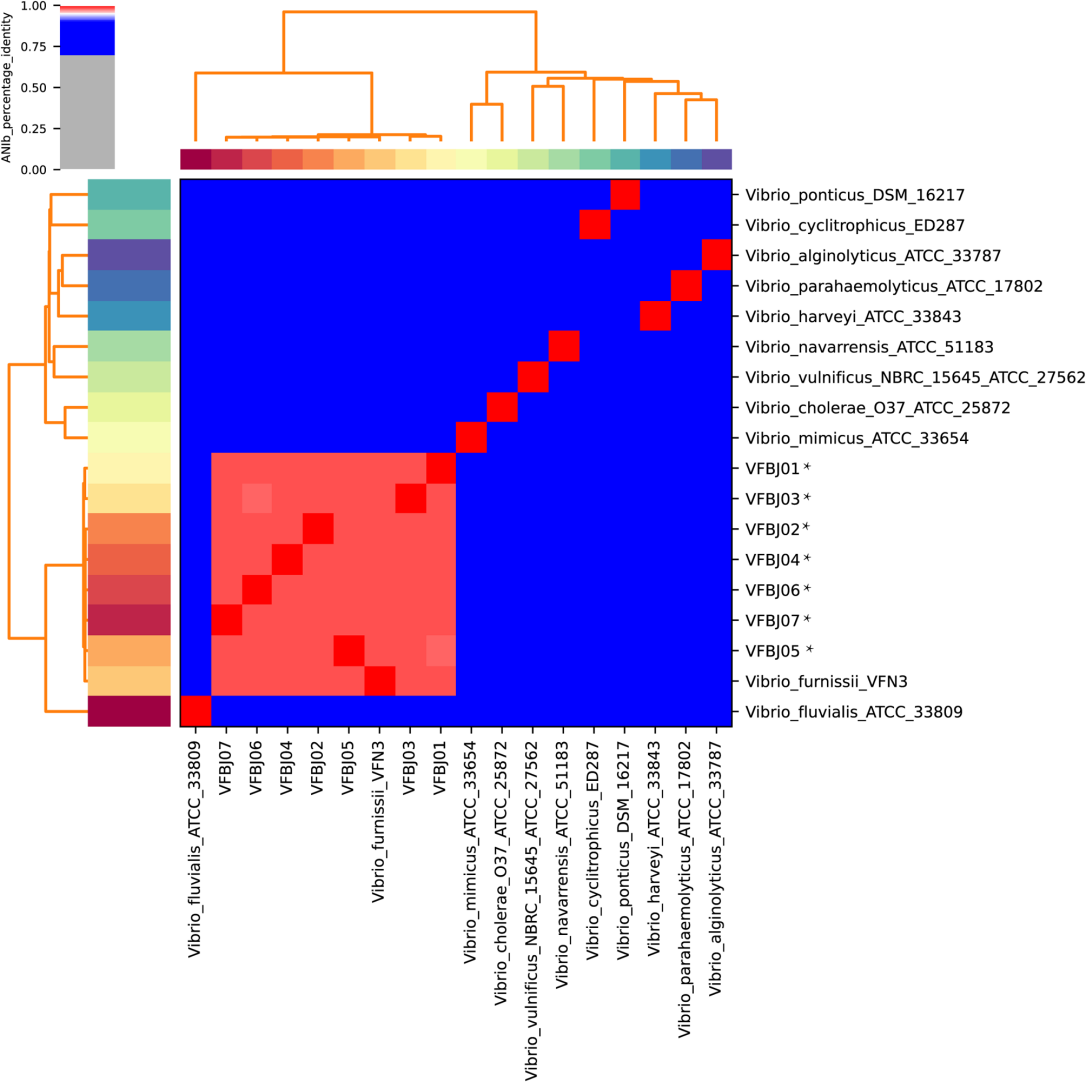
Average nucleotide identity (ANI) analysis of the genomes of 19 Vibrio strains. The strains sequenced in this study are marked with *

*V. furnissii* was first identified using VITEK MS and had 99.0% identified rate, then identified biochemically using VITEK II. We found that the seven *V. furnissii* strains were misidentified as *V. fluvialis*, at a rate of 85.0–99.0% by VITEK II, so we further identified these strains based on ANI [20]. Dendrograms constructed based on the ANI values (Fig. 1) revealed that these seven strains indeed belonged to the *V. furnissii* species. The ANI values of the seven strains ranged from 98.1 to 99.9%, providing accurate identification at the species level. Further genomic analysis showed that these strains contained chromosomes of 4.80 Mb to 5.11 Mb in length, with GC contents ranging from 50.46 to 50.78%. These strains had 8 to 15 genomic islands and one to five prophages in their genomes (Table 2).

**Table 2:** Genome characteristics of the seven *Vibrio furnissii* strains

Sample ID	Genome size (bp)	Fold-coverage	No. of contigs (> 500 bp)	Sequence GC%	No. of genes	No. of genomic islands	No. of Prophages	No. of tRNA	No. of rRNA	No. of sRNA
VFBJ01	5,109,544	652	7	50.46	4,727	15	2	74	1	16
VFBJ02	4,906,640	679	24	50.75	4,596	8	4	86	7	15
VFBJ03	5,062,567	658	8	50.78	4,725	12	3	61	4	14
VFBJ04	4,904,255	679	25	50.58	4,594	13	2	95	6	21
VFBJ05	4,965,951	671	33	50.56	4,656	15	2	99	8	17
VFBJ06	4,802,519	694	28	50.74	4,489	11	1	71	6	15
VFBJ07	5,016,530	664	44	50.56	4,686	12	5	87	6	20

### Population structure analysis

**Fig. 2:**
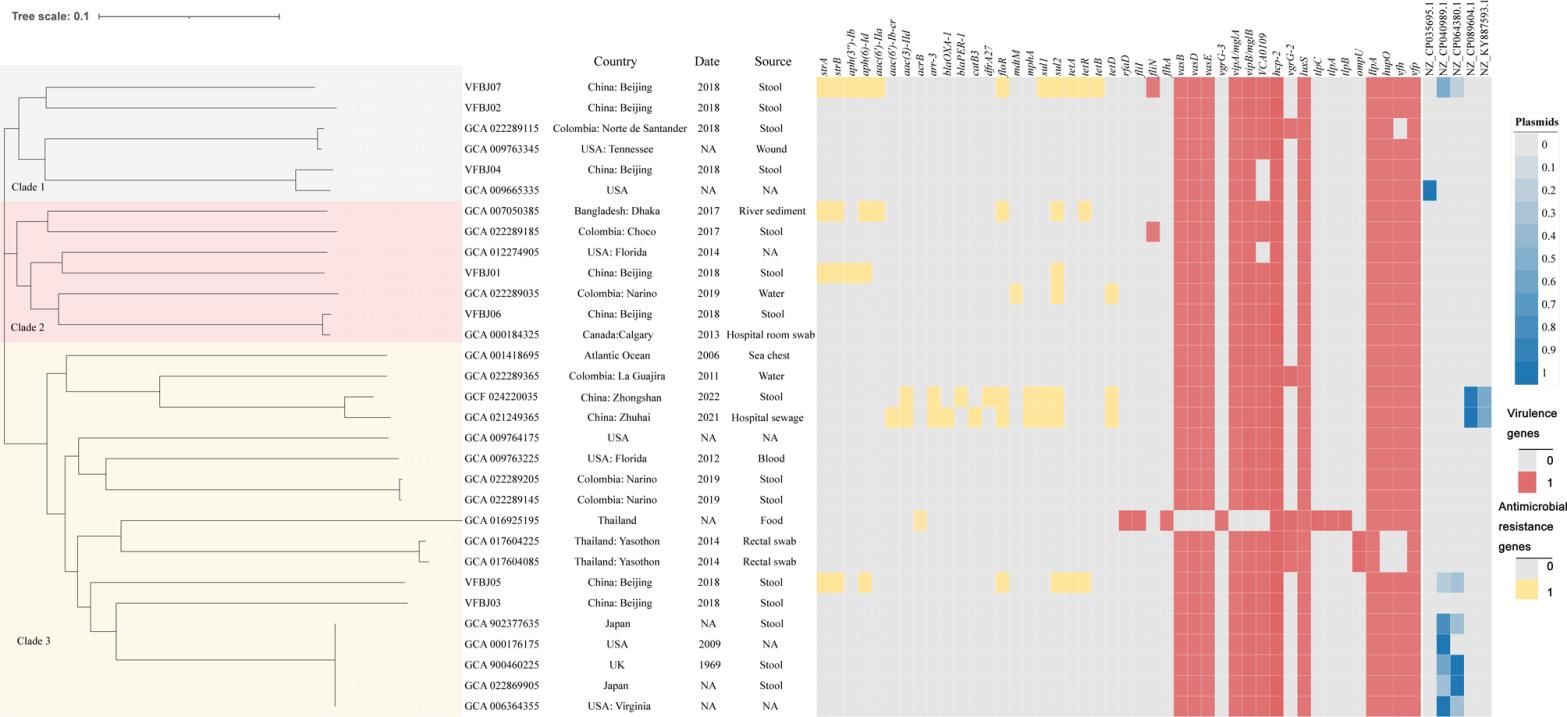
Phylogenetic tree, antimicrobial resistance and virulence gene presence/absence profiles, and plasmid analysis of *Vibrio furnissii* strains, including seven sequences from our study and 24 *V. furnissii* sequences from GenBank, were integrated and rendered using iTOL 3. The isolation year, regions and sources are also shown in the figure. The robustness of tree topologies was evaluated using 1,000 bootstrap replications

Next, we conducted a comparative genome analysis of the seven isolated strains and 24 *V. furnissii* reference sequences from GenBank (Fig. 2). These reference strains were mainly isolated in China, Columbia, the USA, Japan, the UK and Bangladesh during 2006–2022. Phylogenetic trees showed three major clades among the 31 *V. furnissii* strains. Our isolated strains were scattered in these three clades: clade 1 contained VFBJ02, VFBJ04 and VFBJ07; clade 2 contained VFBJ01 and VFBJ06; and clade 3 contained VFBJ03 and VFBJ05. The isolates in the same clade had shorter pairwise evolutionary distances but also occurred in different smaller monophyletic clades, meaning that they did not originate from the same colony.

### Prediction of antimicrobial resistance genes

The antimicrobial resistance gene presence/absence profiles of *V. furnissii* were integrated with phylogenetic trees (Fig. 2). Among our seven isolated strains, VFBJ07 had the largest number of antibiotic resistance genes, including those for aminoglycoside resistance (*strA*, *strB*, *aph(3’’)-Ib* and *aph(6)-Id*), sulphonamide resistance (*sul1* and *sul2*), tetracycline resistance (*tetA*, *tetB* and *tetR*), florfenicol/chloramphenicol resistance (*floR*) and quinolone resistance (*aac(6’)-IIa*). The second largest number of antibiotic resistance genes was found in VFBJ05, which had the same genes mentioned above except for *aph(3’’)-Ib*, *sul1*, *tetB* and *aac(6’)-IIa.* The strain VFBJ01 had the antibiotic resistance genes *strA*, *strB, aph(3’’)-Ib, aph(6)-Id* and *sul2*. In all of the 31 *V. furnissii* sequences, antimicrobial resistance genes were mainly found in strains isolated after 2017, including VFBJ07, VFBJ05, VFBJ01, GCA_007050385 (isolated from river sediments, Bangladesh, 2017), GCA_022289035 (isolated from a water sample, Columbia, 2019), GCF_024220035 (strain 104,486,766 [40], isolated from a stool sample, China, 2022) and GCA_021249365 [19] (isolated from hospital sewage, China, 2021). Among these strains, GCA_021249365 (strain VFN3), submitted by Zhuhai People’s Hospital, had the largest number of antimicrobial resistance genes.

### Susceptibility to antibiotics

The antibiotic resistance profiles of the seven *V. furnissii* isolates to 19 antibiotic agents and the related antibiotic resistance genes are shown in Table 3. High resistance to cefazolin, tetracycline and streptomycin was found in 100.0%, 57.1% and 42.9% of the isolates, respectively, and intermediate resistance to ampicillin/sulbactam and imipenem was found in 85.7% and 85.7% of the isolates, respectively. Four strains – VFBJ01, VFBJ02, VFBJ05 and VFBJ07 – exhibited multi-drug resistance patterns: VFBJ02 was resistant to ampicillin, cefazolin, imipenem, meropenem and tetracycline, and VFBJ01, VFBJ05 and VFBJ07 were resistant to cefazolin, tetracycline and streptomycin.

**Table 3:** Antibiotic susceptibility^a^ patterns and the related antibiotic resistance genes in the seven *Vibrio furnissii* strains

Antibiotic^1^ (µg/mL)	VFBJ01	VFBJ02	VFBJ03	VFBJ04	VFBJ05	VFBJ06	VFBJ07
β-lactam/β-lactamase inhibitor combinations							
AMP	16 (I)	64 (R)	16 (I)	16 (I)	16 (I)	32 (R)	16 (I)
AMS	16/8 (I)	16/8 (I)	16/8 (I)	16/8 (I)	8/4 (S)	16/8 (I)	16/8 (I)
Cephems							
CFZ	16 (R)	32 (R)	16 (R)	16 (R)	8 (R)	32 (R)	32 (R)
CAZ	1 (S)	2 (S)	0.5 (S)	1 (S)	0.5 (S)	1 (S)	0.5 (S)
FEP	1 (S)	8 (I)	1 (S)	1 (S)	1 (S)	1 (S)	1 (S)
AZM	2 (S)	8 (I)	< 1 (S)	2 (S)	2 (S)	1< (S)	< 1 (S)
Aminoglycosides							
AMI	< 4 (S)	< 4 (S)	< 4 (S)	< 4 (S)	< 4 (S)	< 4 (S)	< 4 (S)
GEN	< 1 (S)	< 1 (S)	< 1 (S)	< 1 (S)	< 1 (S)	< 1 (S)	< 1 (S)
STR	> 32 (R)	8 (S)	16 (I)	16 (I)	> 32 (R)	8 (S)	> 32 (R)
Aminoglycoside resistance genes							
*StrA*	+	-	-	-	+	-	+
*StrB*	+	-	-	-	+	-	+
*aph (3’’)-Ib*	+	-	-	-	-	-	+
*aph (6)-Id*	+	-	-	-	+	-	+
Fluoroquinolones							
CIP	0.125 (S)	0.25 (S)	0.06 (S)	< 0.03 (S)	0.125 (S)	0.125 (S)	0.125 (S)
LEV	0.5 (S)	0.5 (S)	0.25 (S)	< 0.125 (S)	0.25 (S)	0.25 (S)	0.25 (S)
Fluoroquinolone resistance gene							
*aac (6’)-IIa*	-	-	-	-	-	-	+
Carbapenems							
IMI	2 (I)	> 8 (R)	2 (I)	2 (I)	2 (I)	2 (I)	2 (I)
MEM	0.25 (S)	4 (R)	0.25 (S)	0.125 (S)	0.125 (S)	0.25 (S)	0.125 (S)
Tetracyclines							
TET	32 (R)	16 (R)	2 (S)	4 (S)	16 (R)	8 (I)	32 (R)
DOX	8 (I)	2 (S)	2 (S)	2 (S)	4 (S)	2 (S)	8 (I)
Tetracycline resistance genes							
*tetA*	-	-	-	-	+	-	+
*tetB*	-	-	-	-	-	-	+
*tetD*	-	-	-	-	-	-	-
*tetR*	-	-	-	-	+	-	+
Phenicols							
CHL	8 (S)	< 2 (S)	< 2 (S)	< 2 (S)	16 (I)	< 2 (S)	8 (S)
Phenicol resistance genes							
*floR*	-	-	-	-	+	-	+
Folate pathway inhibitors							
SUL	128 (S)	64 (S)	< 32 (S)	128 (S)	128 (S)	< 32 (S)	128 (S)
SXT	> 8/152 (R)	< 0.25/4.75 (S)	< 0.25/4.75 (S)	< 0.25/4.75 (S)	< 0.25/4.75 (S)	< 0.25/4.75 (S)	< 0.25/4.75 (S)
SUL resistance genes							
*Sul1*	-	-	-	-	-	-	+
*Sul2*	+	-	-	-	+	-	+
Macrolides							
AZI	< 2 (S)	< 2 (S)	< 2 (S)	< 2 (S)	< 2 (S)	< 2 (S)	< 2 (S)

^1^R: Resistant; I: Intermediate; S: Sensitive; AMP: Ampicillin; AMS: ampicillin/sulbactam; CFZ: cefazolin; CAZ: ceftazidime; FEP: cefepime; AZM: aztreonam; AMI: amikacin; GEN: gentamicin; STR: streptomycin; CIP: ciprofloxacin; LEV: levofloxacin; IMI: imipenem; MEM: meropenem; TET: tetracycline; DOX: doxycycline; CHL: chloramphenicol; SUL: sulphonamides; SXT: trimethoprim–sulfamethoxazole; AZI: azithromycin

^2^ Most breakpoints for defining susceptibility (µg/mL) are based on the CLSI M45-A3 standards for *Vibrio* spp. The breakpoints of AZM and DOX are based on the CLSI M100-ED33 criteria for the Enterobacteriaceae family. The breakpoint of AZI (S ≤ 2) is based on the CLSI M45-A3 standards for *V. cholerae*. The breakpoint of STR (S ≤ 4, I = 8, *R* ≥ 16) is based on the CLSI M45-A3 standards for *Yersinia pestis*

### Plasmid and transposon island analyses

NCBI and Platon searches revealed no plasmid in our seven isolates, with only VFBJ05 and VFBJ07 showing a low coverage, ranging from 20 to 53%, with the sequence of the known plasmid isolated from *V. furnissii* (Fig. 2). Two of the 31 *V. furnissii* sequences, GCF_024220035 [40] and GCA_021249365 [19], contained the same plasmid NZ_CP089604.1 (pVFN3-blaOXA-193 K), an antimicrobial resistance gene-bearing conjugative plasmid [19]. However, whole-genome sequences revealed two plasmids present in VFBJ05 and VFBJ07. The plasmid in VFBJ05 was 85,626 bp long and showed 99.88% identity and 65% coverage with the plasmids CP040989.1 and CP064380.1 reported in *V. furnissii* strain FDAARGOS_777 and *V. furnissii* strain PartQ-Vfurnissii-RM8376, respectively. The plasmid of VFBJ07 was 71,247 bp long and showed 99.90% identity and 79% coverage with the plasmids CP040989.1 and CP064380.1.

**Fig. 3:**
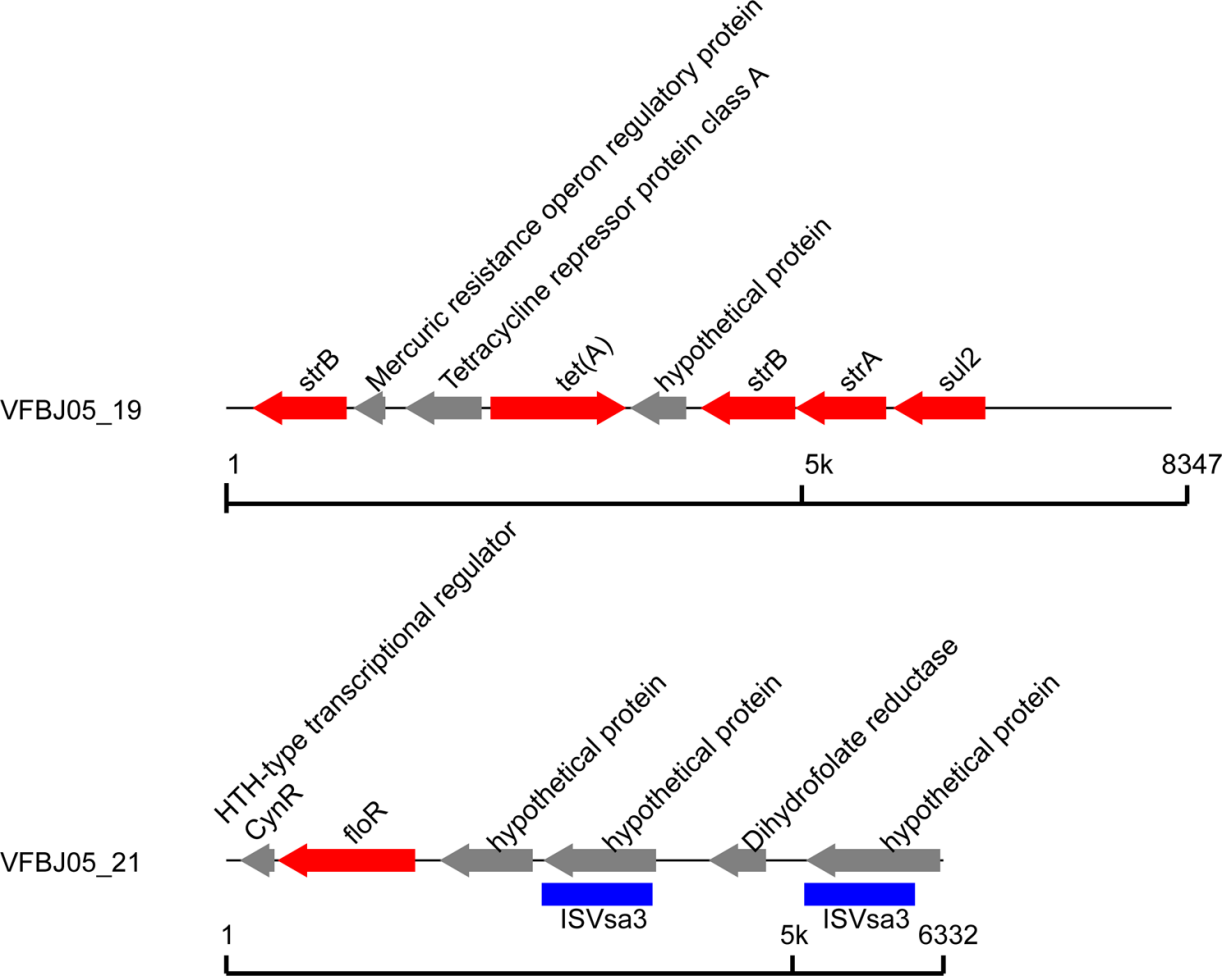
Transposon islands containing the antibiotic resistance genes in VFBJ05. One transposon island contained *strB*, *strA*, *tetA* and *sul2*, and the other contained *floR*

**Fig. 4:**
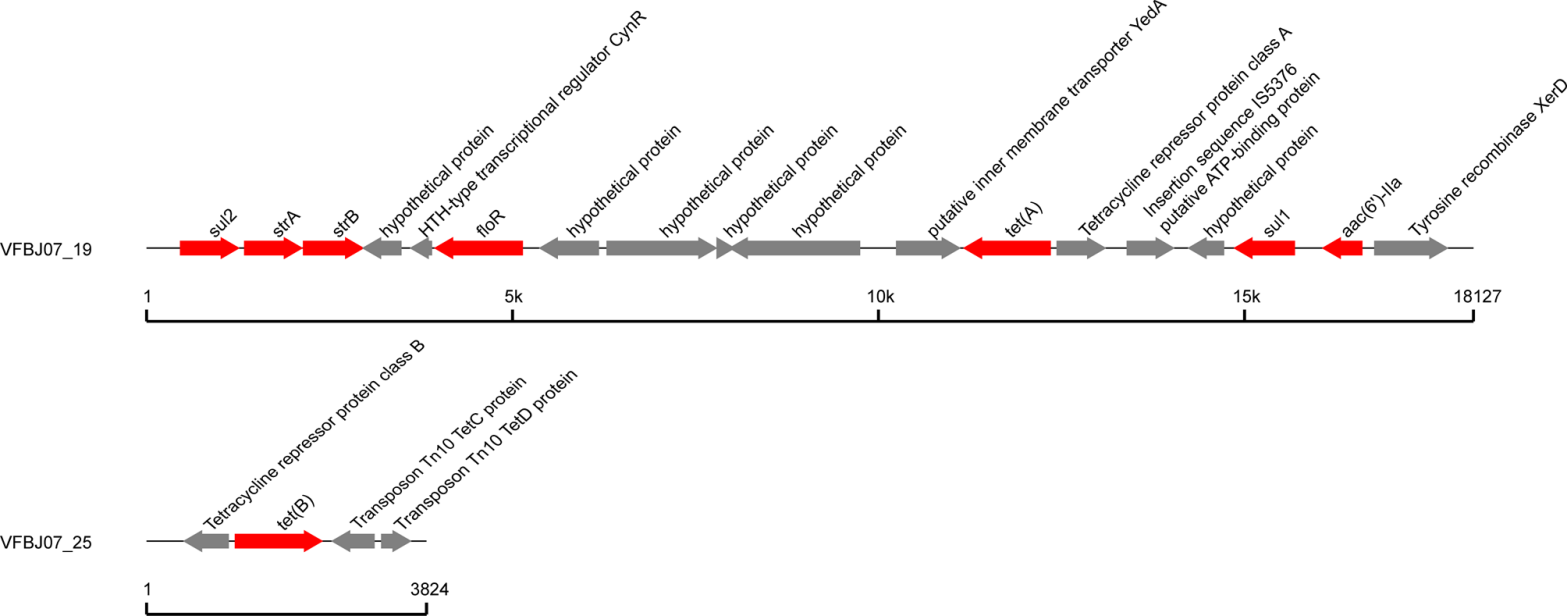
Transposon islands containing the antibiotic resistance genes in VFBJ07. One transposon island contained *strB*, *strA*, *tetA sul1*, *sul2, floR* and *aac(6’)-IIa*, and the other contained *tetB*

Transposon islands were found in VFBJ05 and VFBJ07 and contained antibiotic resistance genes. The transposon islands in VFBJ05 were located on the plasmid, while those in VFBJ07 were located on the chromosome. As shown in Figs. 3 and 4, there were two transposon islands in VFBJ05, one carrying *strB*, *strA*, *tetA* and *sul2* and the other carrying *floR*. There were also two transposon islands in VFBJ07, one carrying *strB*, *strA*, *tetA*, *sul1*, *sul2, floR* and *aac(6’)-IIa* and the other carrying *tetB*.

### Prediction of virulence-associated genes

The virulence gene presence/absence profiles of *V. furnissii* were also integrated with phylogenetic trees (Fig. 2). According to the VFDB, T6SS-related genes (*vipA*, vi*pB*, *vasB*, *vasD*, *vasE* and *hcp-2*), *ilpA* (immunogenic lipoprotein A) [41] and quorum sensing-related gene *luxS* are widespread in *V. furnissii*. As *V. furnissii* was found to be phylogenetically close to *V. fluvialis*, the virulence factor-encoding genes *vfh* (*V. fluvialis* haemolysin) [42], *hupO* (hemin-binding outer membrane protein) [43] and *vfp* (*V. fluvialis* metalloprotease) [44], which widely occur in *V. fluvialis*, were also screened in the genome of *V. furnissii.*

### Virulence phenotypes of *V. furnissii*

**Fig. 5:**
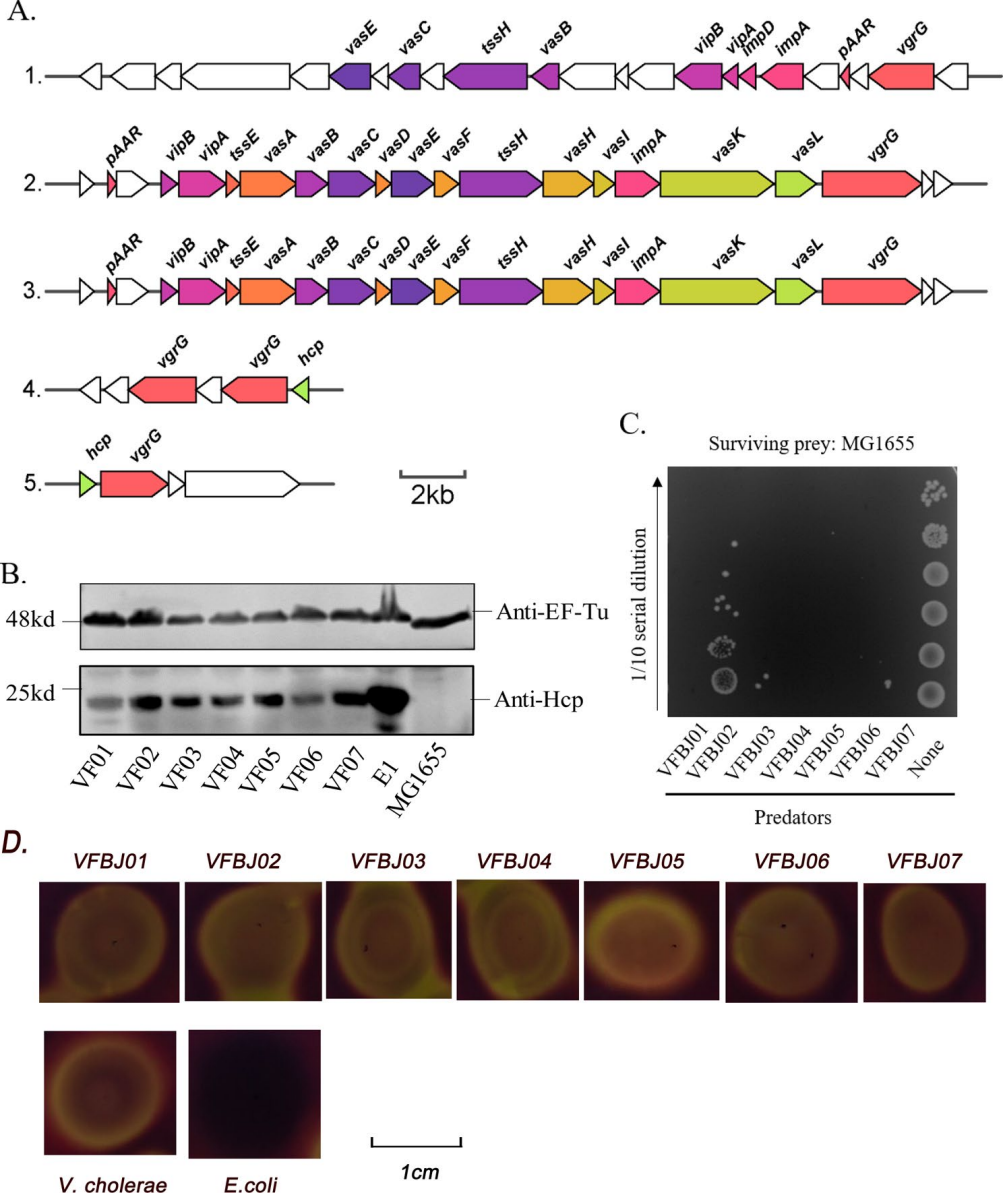
(**A**) The genomic organisation of the structural genes of the T6SS gene clusters from the representative strain VFBJ01. Components encoded by the core genes are shown in various colours. (**B**) Western blot analysis of Hcp expression in seven *Vibrio furnissii* strains. RNA polymerase beta subunit (EF-Tu) was used as the loading control. Original images were included in Supplemental Fig. 1. (**C**) Quantification of *Escherichia coli* MG1655 survival after a T6SS attack by the indicated *V. furnissii* strains. Surviving prey cells (MG1655) were determined by serial dilution and plating on rifampin-containing LB agar plates. Representative images from three independent biological replicates are shown in B and C. (**D**) Quantification of haemolysin expression. Haemolysin assays showed these *V. furnissii* strains grew in large colonies with strong beta-haemolysis on blood agar. Haemolysin-positive control: *V. cholerae* N16961; haemolysin-negative control: *E. coli* MG1655

First, to confirm whether *V. furnissii* encodes the complete T6SS, we analysed the whole genomes of the seven *V. furnissii* strains and found that they all carry genes encoding T6SS elements, including several structural gene clusters and auxiliary gene clusters (Table 4). For example, the representative strain VFBJ01 encodes three core gene clusters and two auxiliary gene clusters (Fig. 5A). To assess whether the T6SS of these strains is active, a western blot assay was used to detect the expression of Hcp, a hallmark of functional T6SS [45]. The results showed that the seven strains constitutively expressed T6SS (Fig. 5B). We further evaluated the effect of their T6SS on inter-bacterial competition. In a competitive killing assay, *E. coli* MG1655 as prey was co-cultured without and with *V. furnissii* strains as predator. The result of this assay showed that the survival of MG1655 co-cultured with *V. furnissii* strains was evidently lower than that of MG1655 co-cultured without *V. furnissii* strains (Fig. 5C). Collectively, these data suggest that *V. furnissii* encodes an activated T6SS and uses it to compete with neighbouring bacterial cells. Further, the haemolysin-related gene *vfh* of *V. furnissii* showed 93–95% similarity to that in *V. fluvialis*, and haemolysin assays showed that *V. furnissii* strains grew in large colonies with strong beta-haemolysis on blood agar, similar to *V. fluvialis* [39] (Fig. 5D).

**Table 4:** Genetic characterisation of the T6SS in *Vibrio furnissii*

Strains		T6SS	Structural cluster	Auxiliary cluster
VFBJ01 VFBI02	+ +		3 2	2 2
VFBJ03 VFBJ04 VFBI05 VFBI06 VFBI07	+ + + + +		2 2 2 2 2	3 2 2 2 2

^+^indicates that this strain harbours T6SS; the number represents the number of T6SS gene clusters

## Discussion

Although *V. furnissii* and *V. fluvialis* are two closely related species, *V. furnissii* differs from *V. fluvialis* in its ability to produce gas through glucose fermentation. However, gas production from glucose fermentation has been reported be variable and thus not reliable for differentiating between the two species [46]. In our study, VITEK MS identified *V. furnissii* strains as accurately as did whole-genome sequencing, and other studies also used MS to confirm *V. furnissii* colonies [7, 10, 46]. These results indicate that MS could be used in clinical laboratories to identify *V. furnissii* quickly and easily.

Many selective-differential media have been developed for isolation of *Vibrio spp.*, however, none of the media developed to date combines the sensitivity to low numbers with the specificity necessary to inhibit growth of other organisms [47]. AMP is frequently added to isolation medium as a selective agent when culturing *Aeromonas* [48]. In this study, as the isolated *V. furnissii* strains caused strong beta haemolysis on blood agar and had intermediate resistance or high resistance to AMP, blood agar plates with 20 µg/mL AMP also helped to isolate more *V. furnissii* compared with only using gentamicin selective medium.

*V. furnissii* has previously been isolated in inland cities such as Beijing, and its total isolation rate in our study was 0.4% (7/1985), which is close to the rate (0.5%) reported in Recife, Brazil [49], an Atlantic seaport city. The reason for such similar rates might be that seafood products are widely consumed worldwide and bacteria of the *Vibrio* genus can contaminate seafood [22]. Although the patients in our study reported no exposure to seawater or seafood, we inferred that seafood might have polluted the food they ate.

Phylogenetic analysis showed that the *V. furnissii* strains were clustered into three main clades, which were not directly related to the isolation location or host source. Our seven *V. furnissii* isolates were in different monophyletic clades in the phylogenetic tree, suggesting that these strains were not associated with outbreaks but were seven independent cases of gastroenteritis.

*V. furnissii* strains express many putative virulence factors, such as a series of virulence factors described in the genome analysis of *V. furnissii* NCTC11218 [20]. In addition, the virulence genes *vfh*, *vfp* and *hupO*, which occur widely in *V. fluvialis*, were also found in *V. furnissii*. VFH expression is associated with strong beta-haemolysis on blood agar [39] and can induce interleukin-1β secretion through the activation of the NLRP3 inflammasome [42]. Given the high similarity of *vfh* between *V. fluvialis* and *V. furnissii*, the haemolysin characterisation of *V. furnissii* was also likely to be related to *vfh*. VFP is a metalloprotease that exhibits haemagglutinating, permeability-enhancing, haemorrhagic and proteolytic activities [43]. HupO, an iron-regulated hemin-binding outer membrane protein, stimulates haemolysin production and resistance to oxidative stress [44]. *V. furnissii* also showed the presence of IlpA, a potent immunogenic lipoprotein that triggers cytokine production in human monocytes by activating Toll-like receptor 2 in *V. vulnificus* [41]. T6SS was first identified in *V. cholerae* [50] and *Pseudomonas aeruginosa* [51]. To date, it has been shown to be widely distributed in approximately 25% of all gram-negative bacteria [51]. T6SS can mediate *V. cholerae* infection in humans [50], and the T6SS of *V. fluvialis* is important for interbacterial competition [52]. In our study, *V. furnissii* was found to express functional T6SS.

*V. furnissii* strains were resistant to ampicillin (85.7%) and cephalothin (21.4%), a first-generation cephalosporin, in a Peru survey in 1995 [8]. Some case reports have also reported that their *V. furnissii* isolates were sensitive to most antibiotics except ampicillin [7, 9, 10]. In comparison, the *V. furnissii* strains in our study showed higher resistance to streptomycin, tetracycline and cefazolin (a first-generation cephalosporin), as well as high rates of intermediate resistance to ampicillin/sulbactam and imipenem. VFBJ02 was also resistant to imipenem and meropenem, both of which are carbapenems, although carbapenem resistance-related genes were not detected. This suggests the presence of an undiscovered carbapenem-resistant mechanism in *V. furnissii*. Imipenem is thus not recommended to treat *V. furnissii* infections. The CDC recommends combination therapy with doxycycline and ceftazidime to treat *V. vulnificus* infection [53]. Although antimicrobial therapy for *V. furnissii* infection has not yet been established, our results suggest that fluoroquinolones and third-generation cephalosporins, such as ceftazidime and doxycycline, are effective at treating *V. furnissii* infection.

Except for the streptomycin resistance shown by VFBJ01, VFBJ05 and VFBJ07 being completely consistent with the presence of *strA*, *strB*, *aph(3’’)-Ib* and *aph(6)-Id* genes, the antibiotic susceptibility patterns of the seven *V. furnissii* strains were partly consistent with the presence of other antibiotic resistance genes. For example, although VFBJ01, VFBJ05 and VFBJ07 carried *sul* genes, their sensitivity to sulphonamides only slightly declined. Similarly, prior studies have shown that resistance genes do not completely correlate with phenotypic resistance [54, 56]. In the present study, the tetracycline resistance of VFBJ05 and VFBJ07 was consistent with the positive detection of *tetA*, *tetB* and *tetR*; however, VFBJ01 and VFBJ02 were also resistant to tetracycline despite carrying no *tet* genes. This finding suggests that other unknown genes influence the relationship between the genotype and phenotype of tetracycline resistance. In addition, although VFBJ07 carried *aac(6’)-IIa*, its fluoroquinolone resistance level was only slightly raised and it remained sensitive to fluoroquinolones. The reason may be that several resistance mechanisms together contribute to fluoroquinolone resistance, including mutations in the quinolone resistance-determining genetic regions and plasmid-mediated quinolone resistance [57]. Ultimately, although the detection and bioinformatic analysis of antibiotic resistance genes cannot completely replace antibiotic susceptibility tests, the existence of antibiotic resistance genes might mean that the strains’ sensitivity to the corresponding antibiotics is declining, thus providing useful guidance for infectious disease treatment.

Several mobile genetic elements, such as insertion sequences, transposons and gene cassettes/integrons, can move within or between DNA molecules and transfer between bacterial cells [58]. GCA_021249365 was isolated from hospital sewage in Zhuhai, Guangdong province, China [19], and GCF_024220035 [40] was isolated from the stool sample of a 63-year-old man in Zhongshan, Guangdong province. These two strains were quite close on the phylogenetic tree, indicating that they might have originated from the same colony. The two strains also possessed the same antimicrobial resistance gene-bearing conjugative plasmid. In our samples, antibiotic resistance genes occurred on transposon islands in VFBJ05 and VFBJ07. The existence of transposon islands carrying antimicrobial resistance indicates that these strains may greatly increase the spread of drug resistance among clinical isolates through the process of infection, especially VFBJ05, in which transposon islands are located on a plasmid.

In conclusion, we found that diarrhoea associated with *V. furnissii* infection occurred sporadically and was more common than expected in the summer in Beijing, China. Fluoroquinolones and third-generation cephalosporins, such as ceftazidime and doxycycline, may be effective at treating *V. furnissii* infection. Two strains – VFBJ05 and VFBJ07 – were found to carry transposon islands containing antibiotic resistance genes. *V. furnissii* had unique virulence characteristics indicated mainly by the presence of T6SS and haemolysis. Overall, the results contribute to our understanding of the bioinformatic and clinical features of *V. furnissii* infections. Future academic and clinical efforts should focus on continual and improved laboratory-based surveillance to prevent and control *V. furnissii* infections and antibiotic resistance gene dissemination.

### Electronic supplementary material

Below is the link to the electronic supplementary material.

Supplementary Material 1

Supplementary Material 2

Supplementary Material 3

Supplementary Material 4

## Data Availability

The datasets used and/or analysed in the current study are available from the corresponding author upon reasonable request.

## Abbreviations

MALDI-TOF MS: matrix-assisted laser desorption/ionisation time-of-flight mass spectrometry
CLSI: Clinical and Laboratory Standards Institute
VFH: *V. fluvialis* haemolysin
HupO: hemin-binding outer membrane protein
VFP: *V. fluvialis* protease
T6SS: type VI secretion system
