# Supplementary material for: Virulence, antibiotic resistance phenotypes and molecular characterisation of Vibrio furnissii isolates from patients with diarrhoea
Source: BMC Infect Dis. 2024 Apr 19;24:412. doi: 10.1186/s12879-024-09273-5 (PMC11027346; doi:10.1186/s12879-024-09273-5)
Supplement: Supplementary file 1 — Supplementary Material 1 [file 12879_2024_9273_MOESM1_ESM.pdf]

asiaedit
Local Knowledge, Global Talent

Armstrong-Hilton Ltd.
2101 Bayfield Building, 99 Hennessy Road, Wanchai, Hong Kong
Tel: +852 2590-6588
Fax: +852 2856-0735
Web: www.asiaedit.com

Certificate

Certificate Date: 21/09/2023
Reference: AE-44511
Customer Id: 15231

To Whom it May Concern

This is to certify that the document listed below has been edited for English language by the professional academic editing company Armstrong-Hilton Limited.

- Document Name: Virulence, antimicrobial resistance phenotypes and molecular characterisation of Vibrio furnissii isolates from patients with diarrhoea
- Author(s): Yanyan Zhou
- Date: 21/09/2023
- Reference: AE-44511

If you have any questions please contact us at cs@asiaedit.com quoting the reference number above. The authors are free to acknowledge our editing service provided the manuscript has not been altered substantially after the editing.

Armstrong-Hilton has been editing and copywriting for the individuals, departments, schools and faculties of Asia's leading academic institutions for 21 years. For more details please see www.asiaedit.com.

For and on behalf of
Armstrong-Hilton Ltd.

ARMSTRONG-HILTON HONG KONG
EDITING

Armstrong-Hilton Ltd. (Authorized Chop)
