# Supplementary material for: Virulence, antibiotic resistance phenotypes and molecular characterisation of Vibrio furnissii isolates from patients with diarrhoea
Source: BMC Infect Dis. 2024 Apr 19;24:412. doi: 10.1186/s12879-024-09273-5 (PMC11027346; doi:10.1186/s12879-024-09273-5)
Supplement: Supplementary file 2 — Supplementary Material 2 [file 12879_2024_9273_MOESM2_ESM.pdf]

SPRINGER NATURE

Change of authorship request form - Journals (pre-acceptance)

Important information. Please read.

- This form should be used by authors to request any change in authorship (adding/deleting authors) including changes in corresponding authors. This form should not be used for name changes. Please fully complete all sections. Use black ink and block capitals and provide each author's full name with the given name first followed by the family name.

- By signing this declaration, all authors guarantee that the order of the authors are in accordance with their scientific contribution, if applicable as different conventions apply per discipline, and that only authors have been added who made a meaningful contribution to the work.

- Please note, in author collaborations where there is formal agreement for representing the collaboration, it is sufficient for the representative or legal guarantor (usually the corresponding author) to complete and sign the Authorship Change Form on behalf of all authors, **next to the added/removed author(s)**. **(Complete Section 3, followed by Section 6.)**
  In author collaborations where there is no formal agreement for representing the collaboration and **there are more than 10 authors**, one may sign for all, provided the signer appends correspondence that attests that each of the authors have agreed to the change **and the added/removed authors sign the form**. **(Complete Section 3, followed by Section 6.)**

- Please note, we cannot investigate or mediate any authorship disputes. If you are unable to obtain agreement from all authors (including those who you wish to be removed) you must refer the matter to your institution(s) for investigation. Please inform us if you need to do this.

- If you are not able to return a fully completed form within **30 days** of the date that it was sent to the author requesting the change, we may have to withdraw your manuscript. We cannot publish manuscripts where authorship has not been agreed by all authors (including those who have been removed).

- Incomplete forms will be rejected.

- Please return/upload this form, fully completed, to the Journals Editorial Office. The Journal and/or Publisher will consider the information you have provided to decide whether to approve the proposed change in authorship. We may decide to contact your institution for more information or undertake a further investigation, if appropriate, before making a final decision.

SPRINGER NATURE Springer Nature is one of the world's leading global research, educational and professional publishers, created in May 2015 through the combination of Nature Publishing Group, Palgrave Macmillan, Macmillan Education and Springer Science+Business Media.

SPRINGER NATURE

Change of authorship request form - Journals (pre-acceptance)

Section 1: Please provide the current title of manuscript

Manuscript ID no.: 90acee67-800f-4ef3-8ea3-f9fc06c321fb v2.1

Title: Virulence, antibiotic resistance phenotypes and molecular characterisation of Vibrio furnissii isolates from patients with diarrhoea

Section 2: Please provide the previous authorship, in the order shown on the manuscript before the changes were introduced. Please indicate the corresponding author by adding (CA) behind the name.

	First name(s)	Family name	ORCID or SCOPUS id, if available
1st author	Yan-yan	Zhou	
2nd author	Li	Yu	
3rd author	Liu	Ming	
4th author	Wei-li	Liang	
5th author	Zheng-peng	Li	
6th author	Zheng	Nan	
7th author	Biao	Kan	
8th author			
9th author			
10th author			

Please use an additional sheet if there are more than 10 authors.

SPRINGER NATURE Springer Nature is one of the world's leading global research, educational and professional publishers, created in May 2015 through the combination of Nature Publishing Group, Palgrave Macmillan, Macmillan Education and Springer Science+Business Media.

Change of authorship request form - Journals (pre-acceptance)

Section 3: Please provide a justification for change. Please use this section to explain your reasons for changing the authorship of your manuscript, e.g. what necessitated the change in authorship? Please refer to the (journal) policy pages for more information about authorship. Please explain why omitted authors were not originally included and/or why authors were removed on the submitted manuscript.

"Ming Liu" was written "Liu Ming" by mistake in our previous manuscript because I confused the order of first name and family name, and "Ming Liu" and "Liu Ming" is the same person. "Zhenpeng Li" was written "Zheng-peng Li" by mistake in our previous manuscript because I confused the front and back nasal sounds in pinyin, "Zhenpeng Li" and "Zheng-peng Li" is also the same person.

Section 4: Proposed new authorship. Please provide your new authorship list in the order you would like it to appear on the manuscript. Please indicate the corresponding author by adding (CA) behind the name. If the Corresponding Author has changed, please indicate the reason under section 3.

	First name(s)	Family name (this name will appear in full on the final publication and will be searchable in various abstract and indexing databases)	Affiliated institute	E-mail address
1st author	Yanyan	Zhou	Beijing Friendship Hospital, China	zhouyanyan1216@163.com
2nd author	Li	Yu	Beijing Center for Disease Prevention and Control, China	wersdu@163.com
3rd author	Ming	Liu	National Institute for Communicable Disease Control and Prevention, China	lium@sustech.edu.cn
4th author	Weili	Liang	National Institute for Communicable Disease Control and Prevention, China	liangweili@icdc.cn
5th author	Zhenpeng	Li	National Institute for Communicable Disease Control and Prevention, China	zpli@qq.com
6th author	Zheng	Nan	Beijing Friendship Hospital, China	nanzheng0127@163.com
7th author	Biao	Kan **(CA)**	National Institute for Communicable Disease Control and Prevention, China	kanbiao@icdc.cn
8th author				

Please use an additional sheet if there are more than 10 authors.

SPRINGER NATURE Springer Nature is one of the world's leading global research, educational and professional publishers, created in May 2015 through the combination of Nature Publishing Group, Palgrave Macmillan, Macmillan Education and Springer Science+Business Media.

Change of authorship request form - Journals (pre-acceptance)

Section 5: Author contribution, Acknowledgement and Disclosures. Please use this section to provide a new disclosure statement and, if appropriate, acknowledge any contributors who have been removed as authors and ensure you state what contribution any new authors made (if applicable per the journal or book (series) policy). Please ensure these are updated in your manuscript - after approval of the change(s) - as our production department will not transfer the information in this form to your manuscript.

New acknowledgements: Not applicable

New Disclosures (financial and non-financial interests, funding): Not applicable

New Author Contributions statement (if applicable per the journal policy): Not applicable

State 'Not applicable' if there are no new authors.

Change of authorship request form - Journals (pre-acceptance)

Section 6: Declaration of agreement. *All* authors, unchanged, new and removed *must* sign this declaration.
(NB: Please print the form, (docu)-sign and return/upload a scanned copy. Please note that signatures that have been inserted as an image file are acceptable as long as it is handwritten. Typed names in the signature box are unacceptable.) *** Please delete as appropriate. Delete all of the bold if you were on the original authorship list and are remaining as an author.**

	First name	Family name		Signature	Date
1st author	Yanyan	Zhou	I agree to the proposed new authorship shown in section 4 and the proposed change in corresponding author	Yanyan Zhou	2024.3.16
2nd author	Li	Yu	I agree to the proposed new authorship shown in section 4 and the proposed change in corresponding author	Li Yu	2024.3.16
3rd author	Ming	Liu	I agree to the proposed new authorship shown in section 4 and the proposed change in corresponding author	ming Liu	2024.3.16
4th authors	Weili	Liang	I agree to the proposed new authorship shown in section 4 and the proposedchange in corresponding author	Weiliang	2024.3.16
5th author	Zhenpeng	Li	I agree to the proposed new authorship shown in section 4 and the proposed change in corresponding author	zhenpeng Li	2024.3.16
6th author	Zheng	Nan	I agree to the proposed new authorship shown in section 4 and the proposed change in corresponding author	Zheng Nan	2024.3.16
7th author	Biao	Kan	I agree to the proposed new authorship shown in section 4 and the proposed change in corresponding author	[illegible]	2024.3.16

SPRINGER NATURE Springer Nature is one of the world's leading global research, educational and professional publishers, created in May 2015 through the combination of Nature Publishing Group, Palgrave Macmillan, Macmillan Education and Springer Science+Business Media.

Change of authorship request form - Journals (pre-acceptance)

	First name	Family name		Signature	Date
8th author			I agree to the proposed new authorship shown in section 4 /**and the addition/removal*of my name to the authorship list** /and the proposed change in corresponding author		
9th author			I agree to the proposed new authorship shown in section 4 /**and the addition/removal*of my name to the authorship list** /and the proposed change in corresponding author		
10th author			I agree to the proposed new authorship shown in section 4 /**and the addition/removal*of my name to the authorship list** /and the proposed change in corresponding author		

Please use an additional sheet if there are more than 10 authors.

In case of author collaborations with formal agreement:

	Name of consortium/consortia	First name	Family name		Signature	Date
Representative/legal guarantor				I agree to the proposed new authorship shown in section 4 /**and the addition/removal*of my name to the authorship list** /and the proposed change in corresponding author		

Both added/removed authors should complete the information in the first table under Section 6.

SPRINGER NATURE Springer Nature is one of the world's leading global research, educational and professional publishers, created in May 2015 through the combination of Nature Publishing Group, Palgrave Macmillan, Macmillan Education and Springer Science+Business Media.

Change of authorship request form - Journals (pre-acceptance)

---- End of form ----

SPRINGER NATURE Springer Nature is one of the world's leading global research, educational and professional publishers, created in May 2015 through the combination of Nature Publishing Group, Palgrave Macmillan, Macmillan Education and Springer Science+Business Media.
