# Supplementary material for: Virulence, antibiotic resistance phenotypes and molecular characterisation of Vibrio furnissii isolates from patients with diarrhoea
Source: BMC Infect Dis. 2024 Apr 19;24:412. doi: 10.1186/s12879-024-09273-5 (PMC11027346; doi:10.1186/s12879-024-09273-5)
Supplement: Supplementary file 3 — Supplementary Material 3 [file 12879_2024_9273_MOESM3_ESM.pdf]

首都医科大学附属北京友谊医院

医学伦理委员会伦理审查批件

批件号：2017-P2-095-01

<table>
<tr><td>试验项目名称</td><td colspan="5">临床分离气单胞菌MLPA分型、毒力基因及耐药特征分析</td></tr>
<tr><td>申办单位</td><td colspan="5">首都医科大学附属北京友谊医院</td></tr>
<tr><td>项目来源</td><td colspan="5">首都卫生发展科研专项项目</td></tr>
<tr><td>本院申请部门</td><td>检验科</td><td>承担责任</td><td>主要责任</td><td>项目负责人</td><td>周妍妍</td></tr>
<tr><td>主要审查文件</td><td colspan="5">文件名称 ｜ 版本号/版本日期
递交信 ｜
初始审查申请 ｜
临床研究方案 ｜ V1.0/2017.8.15
项目概况表（科研项目用） ｜
主要研究者专业履历 ｜</td></tr>
<tr><td>伦理委员会声明</td><td colspan="5">*本伦理委员会严格按照ICH/GCP、中国GCP及相关法规组成和工作。
*本伦理委员会的组成和工作相对独立。</td></tr>
<tr><td>审查方式</td><td colspan="5">■会议审查 □快速审查 ■初始审查 □跟踪审查</td></tr>
<tr><td>审查时间</td><td colspan="2">2017-8-31</td><td>会议地点</td><td colspan="2">九层三会</td></tr>
<tr><td>审查委员</td><td colspan="5">见会议签到表。投票7人，同意7人，作必要修正后同意0人，作必要修正后重审0人，不同意0人，终止或暂停已批准的试验0人。回避0人。</td></tr>
<tr><td colspan="6">审查意见：
根据卫生部《涉及人的生物医学研究伦理审查办法（试行）》(2007)、SFDA《药物临床试验质量管理规范(2003)》、《医疗器械临床试验规定(2004)》、WMA《赫尔辛基宣言》和CIOMS《人体生物医学研究国际道德指南》的伦理原则，经本伦理委员会审查：
同意按所批准的临床研究方案、知情同意书、招募材料等开展本项研究。
备注：
1.本项临床试验应当在伦理委员会同意进行之日起1年内实施。逾期未实施的，本批件自行废止。
2. 修正后同意/复审项目，应将修正后文件及时反馈给伦理委员会，以便签署意见或安排复审。
3. 在试验实施过程中，如需对研究方案、知情同意书等文件做任何修改，应及时向本伦理委员会提交修改申请，经重新审查，获得批准后方可执行。
4.发生严重不良事件及可能影响风险/受益比的任何事件和新信息须及时报告伦理委员会。
5.定期/年度跟踪审查项目，于到期前1个月（无论试验开始与否）提交定期跟踪审查申请。</td></tr>
</table>

6. 如有不依从/违背方案或暂停/提前终止的试验项目，应及时以书面文件告知本伦理委员会。<br>7. 临床试验结束后，须及时向伦理委员会提交结题报告。请申请人提交研究完成报告。完成临床研究，请申请人提交研究完成报告。	
年度/定期跟踪审查频率	12 个月
有效期	12 个月
主任委员签字：<br>日 期：2017 年 9 月 1 日	伦理委员会（盖章）：

伦理委员会地址：北京市西城区永安路 95 号（邮编：100050） 联系人：崔焱 电话/传真：010-63139017

首都医科大学附属北京友谊医院

医学伦理委员会

伦理审批文件清单

2017-P2-095-01

研究项目：临床分离气单胞菌 MLPA 分型、毒力基因及耐药特征分析

会议审批文件：

文件名称	版本号/版本日期
递交信	
初始审查申请	
临床研究方案	V1.0/2017.8.15
项目概况表（科研项目用）	
主要研究者专业履历	

首都医科大学附属北京友谊医院

医学伦理委员会

日 期：2017.9.1

文件编号：AF/SC-02/03.0

首都医科大学附属北京友谊医院伦理委员会 会议签到表

姓名	性别	伦理委员会职务	所在单位及职务		签名	日期	是否回避	
							是	否
张建	男	主任委员	北京友谊医院	医院管理. 儿外科	张建	2017.8.31		✓
尤红	女	副主任委员	北京友谊医院	科研管理. 消化内科	[illegible]	2017.8.31		✓
史丽敏	女	委员	北京友谊医院	药学	[illegible]	2017.8.31		✓
徐芳	女	委员	北京友谊医院	妇产科	徐芳	2017.8.31		✓
马莉	女	委员	北京友谊医院	儿科学				
孙燕	女	委员	北京友谊医院	护理学	[illegible]	2017.8.31		✓
邓利强	男	委员	北京市华卫律师事务所	律师	[illegible]	2017.8.31		✓
江欢	男	委员	首都医科大学	教师				
崔焱	女	委员秘书	北京友谊医院	肝脏病学	[illegible]	2017.8.31		✓

本名单的适用期从 2015 年 6 月 1 日开始。若伦理委员会委员组成发生变化，名单将及时更新。

首都医科大学附属北京友谊医院 医学伦理委员会
