# Supplementary material for: Virulence, antibiotic resistance phenotypes and molecular characterisation of Vibrio furnissii isolates from patients with diarrhoea
Source: BMC Infect Dis. 2024 Apr 19;24:412. doi: 10.1186/s12879-024-09273-5 (PMC11027346; doi:10.1186/s12879-024-09273-5)

**Supplemental Figure**

Supplemental Fig. 1**:** Original images of western blot . Red boxes indicated interest protein regions, one of which was shown in Figure 5B.


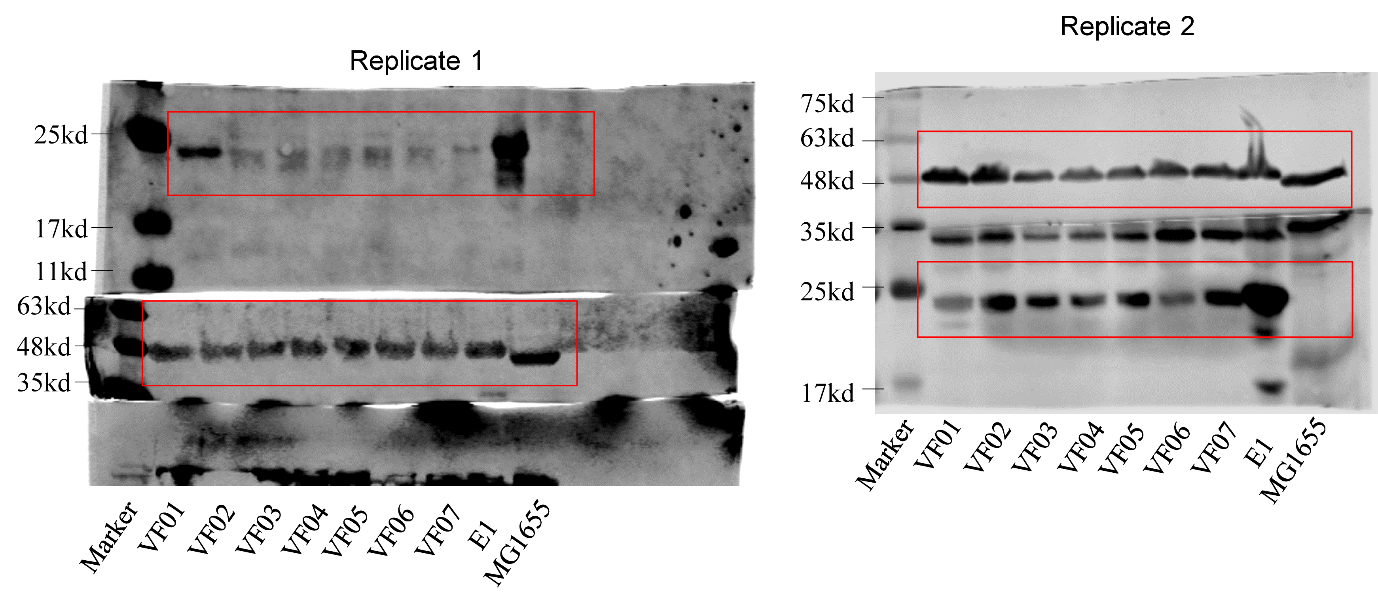

Supplement: Supplementary file 4 — Supplementary Material 4 [file 12879_2024_9273_MOESM4_ESM.docx]
